# Rifampin-resistant Meningococcal Disease

**DOI:** 10.3201/eid1106.050143

**Published:** 2005-06

**Authors:** Jean Rainbow, Elizabeth Cebelinski, Joanne Bartkus, Anita Glennen, Dave Boxrud, Ruth Lynfield

**Affiliations:** * Minnesota Department of Health, Minneapolis, Minnesota, USA

**Keywords:** Antibiotic resistance, Neisseria meningitidis, meningococcal infections, chemoprophylaxis, rifampin, RNA polymerase beta subunit

## Abstract

Rifampin-resistant meningococcal disease occurred in a child who had completed rifampin chemoprophylaxis for exposure to a sibling with meningococcemia. Susceptibility testing of 331 case isolates found only 1 other case of rifampin-resistant disease in Minnesota, USA, during 11 years of statewide surveillance. Point mutations in the RNA polymerase β subunit (*rpoB*) gene were found in isolates from each rifampin-resistant case-patient.

Chemoprophylaxis is recommended for close contacts of persons with invasive meningococcal disease to prevent secondary cases. In the 1960s, rifampin replaced sulfonamides as the recommended agent for chemoprophylaxis of household members and other close contacts of persons with invasive meningococcal disease when sulfonamide-resistant meningococci became common (1). In recent years, ciprofloxacin and ceftriaxone have been established as acceptable alternatives to rifampin for prophylaxis of meningococcal disease. However, rifampin remains a popular choice due to its low cost, ease of administration, and well-established record among infants and children.

Pharyngeal colonization with rifampin-resistant meningococci following chemoprophylaxis with rifampin of persons exposed to meningococcal disease was documented soon after treatment was initiated (2) and has continued to be observed over time (3). However, although rifampin has been used routinely worldwide for more than 30 years, few cases of rifampin-resistant meningococcal isolates in cases of invasive disease have been reported (4–7), and reports of only 3 instances in the United States could be found (8–10).

Rifampin targets the β subunit of DNA-directed RNA polymerase by inhibiting extension of the RNA strand. The β subunit is encoded by the *rpoB* gene. Previous studies have demonstrated that one of the mechanisms of rifampin resistance in *Neisseria meningitidis* is associated with single point mutations of the *rpoB* gene that result in amino acid substitutions (11–13). The data presented in this study confirm the rapid development of rifampin resistance upon exposure of meningococci to rifampin as a result of point mutations in the *rpoB* gene.

## The Study

Cases of invasive meningococcal disease in Minnesota residents are required to be reported to the Minnesota Department of Health (MDH). Laboratories throughout the state routinely submit isolates from patients with this disease to the MDH Public Health Laboratory, where they are serogrouped by slide agglutination (Difco, Detroit, MI, USA). In 1995, the MDH began routinely testing antimicrobial susceptibilities on meningococcal isolates and retrospectively conducted susceptibility testing on all available meningococcal isolates that had been submitted since 1993.

Antimicrobial susceptibilities were determined by using broth microdilution. Panels contained cation-adjusted Mueller-Hinton broth with 2%–5% lysed horse blood (PML Microbiologicals, Wilsonville, OR, USA) and were incubated at 35°C in CO_2_ for 20–24 h. An Etest (AB Biodisk, Solna, Sweden) was also used for isolates that demonstrated resistance to further quantify degree of resistance. MIC breakpoints have recently been established by the Clinical and Laboratory Standards Institute for *N. meningitidis* (14). An MIC ≥2 μg/mL is considered resistant to rifampin.

Molecular subtyping of the sibling isolates was done by pulsed-field gel electrophoresis (PFGE) as described previously (15). The *rpoB* genes from rifampin-resistant and rifampin-sensitive isolates (Table 1) were amplified by polymerase chain reaction and sequenced by using primers described previously (13). DNA and peptide sequences were analyzed with BioNumerics (Applied Maths, Austin, TX, USA) and Vector NTI Suite (InforMax, North Bethesda, MD, USA).

**Table 1:** Rifampin phenotype and genotype of Neisseria meningitidis isolates

Strain	Description	Rifampin MIC (μg/mL)	Amino acid change*
MDH02-2342	Sporadic rifampin-susceptible serogroup C case isolate	0.004†	None (WT)
MDH02-2271	Sporadic rifampin-susceptible serogroup B case isolate	<0.002†	None (WT)
MDH97-498	Isolate from sporadic rifampin-resistant serogroup B case in 1996	>4,† >32‡	His_552_Tyr§
MDH02-2398	First sibling's isolate: rifampin susceptible, serogroup C	0.008†	None (WT)
MDH02-2408	Second sibling's isolate: rifampin resistant, serogroup C	>1,† >32‡	Ser_548_Phe§

*WT, wildtype. †Determined by broth microdilution. ‡Determined by Etest. §Numbering based on the entire *N. meningitidis rpoB* gene (GenBank accession no. Z54353). Accession numbers of isolate sequences submitted to GenBank: MDH02-2342 (AY746965), MDH02-2271 (AY746964), MDH97-498 (AY746963), MDH02-2398 (AY746966), MDH02-2408 (AY746967).

The first known case of rifampin-resistant invasive meningococcal disease in Minnesota occurred in 1996. A 5-month-old infant had a clinical syndrome consistent with meningococcemia. He was hospitalized for 10 days, received antimicrobial drug therapy, and survived. By Etest, his serogroup B *N. meningitidis* isolate had a rifampin MIC ≥32 μg/mL. This was a sporadic case with no apparent links to any other previous or subsequent cases.

In 2002, fever, vomiting, and irritability developed in a 2-month-old infant, followed 12 hours later by labored breathing and a generalized rash. She was taken to a clinic where she experienced cardiac arrest and underwent cardiopulmonary resuscitation. She was transferred to a nearby emergency room where she died ≈1 hour later. Meningococcemia was suspected and household members were given prescriptions for rifampin. Waterhouse-Friderichsen syndrome was noted on autopsy, and *N. meningitidis* was isolated from a swab of brain tissue. Three days after the death of the case-patient and 1 day after completing a 2-day course of rifampin, a fever and lethargy developed in the case-patient's 6-year-old sister. Blood cultures were obtained and she was hospitalized, given antimicrobial drug treatment (ceftriaxone), and observed. No cerebrospinal fluid was collected. Blood cultures were subsequently positive for *N. meningitidis*. She responded to ceftriaxone and continued treatment as an outpatient after a short hospitalization. Household contacts, along with other close contacts of the 6-year-old girl, again received chemoprophylaxis. It was recommended that adults be treated with ciprofloxacin and children be treated with ceftriaxone because of concerns that 1 or both siblings could have had rifampin-resistant meningococcal infections. No additional related cases were identified over the following weeks.

Isolates from both siblings were identified as serogroup C. The PFGE patterns were indistinguishable and had, in fact, the most common PFGE pattern seen for that serogroup in Minnesota. Antimicrobial susceptibility testing showed that the isolate from the case-patient was susceptible to ceftriaxone, penicillin, chloramphenicol, ciprofloxacin, and rifampin. The MIC for rifampin was 0.008 μg/mL. The isolate from the 6-year-old patient was susceptible to the same drugs, except for rifampin, which had an MIC >1 μg/mL by broth microdilution and an MIC >32 μg/mL by Etest.

A comparison of the nucleotide sequence of the *rpoB* gene of both sibling isolates showed they were identical except for a single nucleotide change. This change resulted in a substitution of serine for phenylalanine at amino acid position 548. This substitution has previously been associated with rifampin resistance in *N. meningitidis* (12).

The PFGE subtype of the isolate from the rifampin-resistant case in 1996 differed from that of the siblings' isolates. Sequencing of the *rpoB* gene from this isolate showed an amino acid substitution of histidine for tyrosine at position 552. This substitution has also been previously associated with rifampin resistance in *N. meningitidis* (Table 1; MDH97-498) (11,13).

Susceptibility results on meningococcal isolates from 1993 to 2003 for other antimicrobial agents are shown in Table 2. Using the newly established breakpoints, we observed that 92% (303/331) of the isolates were susceptible to penicillin, 100% (205/205) were susceptible to ceftriaxone, 100% (331/331) were susceptible to meropenem, 100% (205/205) were susceptible to ciprofloxacin, 100% (331/331) were susceptible to chloramphenicol, and 48% (158/331) were susceptible to trimethoprim-sulfamethoxazole.

**Table 2:** Antimicrobial drug susceptibilities for meningococcal invasive disease *Neisseria meningitidis* isolates, Minnesota, USA, 1993–2003*

Antimicrobial drug susceptibility†	1993	1994	1995	1996	1997	1998	1999	2000	2001	2002	2003
Rifampin	100	100	100	97	100	100	100	100	100	97	100
Ceftriaxone	NA	NA	NA	NA	NA	100	100	100	100	100	100
Ciprofloxacin	NA	NA	NA	NA	NA	100	100	100	100	100	100
Chloramphenicol	100	100	100	100	100	100	100	100	100	100	100
Meropenem	100	100	100	100	100	100	100	100	100	100	100
Penicillin	92	100	81	89	92	83	96	95	96	86	93
Trimethoprim-sulfamethoxazole	30	55	74	62	32	42	29	36	52	58	66
No. of cases	27	23	30	40	40	36	56	22	27	36	29
No. of isolates tested (%)	13 (48)	11 (48)	27 (90)	37 (93)	38 (95)	36 (100)	55 (98)	22 (100)	27 (100)	36 (100)	29 (100)

*NA, not available. †Values for antimicrobial drugs are % of isolates susceptible by broth microdilution.

## Conclusions

Primary cases of rifampin-resistant meningococcal disease are rare. While more common, secondary cases with rifampin resistance can develop following chemoprophylaxis with rifampin. All *N. meningitidis* isolates tested at MDH were susceptible to ceftriaxone and ciprofloxacin. Ceftriaxone must be given parenterally but is the recommended prophylactic agent for infected pregnant women. According to the 2003 American Academy of Pediatrics Report of the Committee on Infectious Diseases, ciprofloxacin may be used by persons >15 years of age. While few instances of ciprofloxacin resistance have been reported, its widespread use may result in greater resistance in *N. meningitidis* (as has occurred in related pathogens such as *Neisseria gonorrhoeae*) (16,17). Persons receiving chemoprophylaxis should be advised about the potential of meningococcal disease developing, even though they have taken antimicrobial agents as prescribed. If a close contact who has been treated with rifampin becomes ill with meningococcal disease, alternative antimicrobial agents should be used for prophylaxis until rifampin sensitivity of the secondary infection can be established. Although rifampin-resistant meningococcal disease is still rare after 30 years of using rifampin for chemoprophylaxis and ciprofloxacin resistance has rarely been observed, susceptibilities to chemoprophylactic agents should be monitored to ensure that recommendations are sufficiently effective to minimize the occurrence of secondary cases.
